# Medicine, Present and Prospective
*An abstract of the Address in Medicine delivered at the seventy-third annual meeting of the British Medical Association by Henry Maudsley, M.D., F.R.C.P.


**Published:** 1905-08-05

**Authors:** 


### Hospital Clinics.

### MEDICINE, PRESENT AND PROSPECTIVE.*

The present state and prospect of medicine indicate that its work in the future will be mainly to prevent and stop the beginnings of disease. Preventive medicine has two main ends—the one the protection of the body from assaults from without, the other, to obviate the predispositions to disease which lie within the body itself by giving it strength to resist its subtle and ever-active enemies. The first of these is being pursued with success; but the latter receives only inadequate attention, being thrown into the background by the eager quest for the microbe. Such predispositions to disease, however, exist, though the study of different types of constitution and of their respective qualities and disease-tendencies has been altogether neglected since the abandonment of the old doctrine of temperaments. Another factor which might be considered with advantage is the effects of the interbreeding of parental disease-tendencies upon the constitution of the offspring. When an individual has a disease unlike that which either of his parents had he still may owe it to them, variations occurring in morbid just as they do in physiological heredity. Such questions as the constitutional dispositions most likely in breeding to conceal a tendency to cancer, to convert a native bias towards insanity into a good evolutional variation, to counteract the probability of unfortunate neuropathic incidents, have a close and obvious bearing on the prevention of some of the most disastrous diseases. Illustrations of the same principle are seen in such incidents as the translation of the epilepsy of one generation into the insanity of the next; and the association, sometimes in alternate sequence, of diabetes and insanity in the same family. When these and numerous allied questions can be answered medical science will be in a position to dictate some wise eugenic rules. Meanwhile trust must be placed in the *vis medicatrix naturæ* to rectify deviations and to bring disorder back to order.

In fortifying the individual body to resist the inroads of disease the most simple means—pure air, clean and proper food, regular and adapted exercise—are the best. But they are not quite all, for they leave out mind. Unruly affections and passions are a hurt to body as well as to mind, so that rules of wise mental culture and exercise are an essential part of the regimen of health.

Another direction from which help might be obtained in the strengthening of the bodily resistance is the study of the motor, sensory, secretory, and nutritional reflexes. Such knowledge would yield much profitable use in the precise application of mechanical measures whereby infirm organs may be sometimes benefited, and also in the application of regulated exercises by which every muscle in the body may be made to contract and every minute vital channel kept open.

The present posture of medicine in relation to tuberculosis recognises the necessity of fortifying the body to resist its outward enemies. The bacilli have been discovered, but it is probably hopeless to expect that they can be absolutely prevented from entering human bodies. Nor has time brought either a protective serum to immunise the body, or an antitoxic serum to extirpate the bacillus in it. Some want of sobriety in statements made on popular platforms has led a large section of the public to conclude that because the bacillus has been discovered phthisis is necessarily curable, the former notion of heredity erroneous, the objection to the marriage of the tuberculous obsolete, and the wise policy to dot the surface of the land freely with sanatoriums. So far as statistics go it may be questioned whether pulmonary phthisis is an eminently curable disease even when treated in special hospitals which rejoice in this distinctive title. Early cases probably recover, provided they are kept sufficiently long; cases in a more advanced stage usually improve, but frequently relapse afterwards; whilst more extreme cases ought not to be sent to these institutions. After all, these results are much the same as those previously observed under any sensible form of treatment. The demand still remains to mend the fit constitutional soil and breeding-ground of the bacilli if these are to perish of inanition for want of human food. Actual tubercle may not be inherited, but the poor constitutional soil inviting and suiting the bacillus still passes from parent to child. And it may be asked whether the estimated national loss placed against the influence of the tubercle bacillus does detract so much from the life-capital of the people as is sometimes suggested.

Another aspect of the body closely related to the prevention of disease is the recognition of the body as a chemical laboratory in which numerous complex processes are in course. Every cell is the seat of such changes, and produces in the performance of its functions toxic substances which are prejudicial to the welfare both of itself and its fellow cells. Hence these must be collected by the blood-stream and carried away to the excretory organs to be discharged from the body. Toxic products again are produced in the alimentary canal, and from this source also self-poisoning may occur. Another example of chemical action modifying health is found in the toxins which are the product of microbic invasion, and some of the results of which may be apparently postponed for many years after the primary infection.

In the discovery of many toxins and antitoxins there may be detected the promise of a more scientific therapeutics, when chemical agents will be used, not empirically, but with scientific precision and for a definite end. Similarly it is to be noted that with every forward step in physics and chemistry proofs accumulate that all vital processes rest at bottom on physical and chemical processes of the same kind as those which take place out of the living body. Further, while between material energy and consciousness there is certainly a gulf, it is not possible in biology to set up a partition

* An abstract of the Address in Medicine delivered at the seventy-third annual meeting of the British Medical Association by Henry Maudsley, M.D., F.R.C.P.

between mind and body, and it is probably not a mere fancy to suggest that a mental atmosphere or effluence emanates from one person to affect another, either soothing sympathetically or irritating antipathically. A just conception of the subtleties of the forces at work in mental action may thus inspire a more advised and methodical use of the resources of the mind to cure the diseases of the body. The recognition of this truth is the grain of accuracy in faith-healing and similar forms of quackery. Sick persons are notably sick in mind and mostly need a mental tonic to stimulate their weakened vitality. In this way tissue-resistance may be increased and the tendency to recovery be heightened. Few drugs are more helpful than hope, more deadly than despair.

Medical science must needs play a large part in the future improvement of the race, for it is certain that, though the problem is a complex and difficult one, there are laws of mental breeding yet to be discovered, and the nature of vice and sin are as capable of scientific study as are the actions of poisons. The saying of Descartes that if mankind is to be perfected the means of perfecting it must be sought in the medical sciences may be accepted as true, and gradually it will become plain that. by the operation of natural law, sound thought, good moral feeling, and devotion to a high ideal are the solid foundations of health and wealth of mind alike in individuals, in families, and in nations.

